# Possible Central Nervous System Infection by SARS Coronavirus

**DOI:** 10.3201/eid1002.030638

**Published:** 2004-02

**Authors:** Kwok-Kwong Lau, Wai-Cho Yu, Chung-Ming Chu, Suet-Ting Lau, Bun Sheng, Kwok-Yung Yuen

**Affiliations:** * Princess Margaret Hospital, Kwai Chung, Hong Kong Special Administrative Region (SAR), China; † United Christian Hospital, Kwun Tong, Hong Kong SAR, China; ‡ Queen Mary Hospital, University of Hong Kong, Hong Kong SAR, China

**Keywords:** coronavirus, severe acute respiratory syndrome, convulsion

## Abstract

On day 22 of illness, generalized tonic-clonic convulsion developed in a 32-year-old woman with severe acute respiratory syndrome (SARS). Cerebrospinal fluid tested positive for SARS coronavirus (SARS-CoV) by reverse transcriptase–polymerase chain reaction. SARS-CoV may have caused an infection in the central nervous system in this patient.

Severe acute respiratory syndrome (SARS) is an acute febrile illness predominantly involving the lungs, and a high proportion of patients die of respiratory failure (*1*). However, the novel coronavirus causing SARS appears not to be confined to the lungs, as suggested by observation of diarrhea (*2*), impaired liver function tests, lymphopenia, and thrombocytopenia (*3*). We report a case of possible involvement of the central nervous system by the SARS-coronavirus (SARS-CoV).

## Case Report

A 32-year-old woman in week 26 of pregnancy, who was previously in good health, was admitted to the hospital on March 29, 2003 with myalgia for 1 week and fever, chills, and rigor for 2 days. She had an unproductive cough and no sore throat. On admission, her temperature was 38.8°C, with chest radiograph showing patchy consolidations over the right upper lobe and both lower lobes. Total leukocyte count was 12.3 × 10^9^/L and lymphocyte count was 1.6 × 10^9^/L. Hemoglobin level, liver and renal function tests, and serum lactate dehydrogenase were normal. Ribavirin (500 mg every 8 hours) and hydrocortisone (100 mg every 8 hours) were administered intravenously. Clinical and radiologic deterioration progressed, and by day 7, mechanical ventilation was begun. Onset of acute renal failure began on day 8, with oliguria and rapidly elevating serum urea and creatinine levels, and a decision was made to terminate the pregnancy. Lower-segment cesarean section was performed on the same day, and a baby girl, appropriate to the gestational age, was born. Mechanical ventilation continued, but renal function continued to deteriorate. She was given piperacillin/tazobactam to cover possible sepsis. On day 10, the serum creatinine level was 504 μmol/L, and the patient was still oliguric, necessitating hemodialysis intermittently from day 10 to day 18. The diuretic phase was administered on day 19, and her renal functions improved progressively.

On day 22, the patient was still on mechanical ventilation and was sedated with an infusion of 30 mg of midazolam plus 30 mg of morphine in 50 mL 5% dextrose solution at 6 mL per hour. During the previous 2 days, she had a low-grade fever. Early that afternoon she had a generalized tonic-clonic convulsion with loss of consciousness and up-rolling eyeballs lasting for 1 minute. She had no neck rigidity and no residual neurologic deficit. Lumbar puncture performed later that day showed an opening pressure of 15 cm of water, with free flow of clear cerebrospinal fluid (CSF). Total CSF protein was 0.38 g/L, and CSF glucose was 5.1 mmol/L, against blood glucose of 6.6 mmol/L. Microscopy showed an erythrocyte count of 20 per mm3 and a leukocyte count of <1 per mm^2^. Gram stain, bacterial cultures, and viral cultures were negative. However, reverse transcriptase–polymerase chain reaction (RT-PCR) on CSF for the SARS-CoV was positive. Serum calcium was 1.96 mmol/L against a serum albumin of 27 g/L, serum magnesium was 0.62 mmol/L (normal range 0.7–1.1 mmol/L), serum sodium was 152 mmol/L (normal range 135–149 mmol/L), serum potassium was 4.0 mmol/L, and serum creatinine was 311 μmol/L. Both the electroencephalogram done on day 39 and magnetic resonance imaging done on day 46 showed no abnormalities. She had no other convulsions.

Renal function and respiratory status continued to improve. She was extubated on day 27 and made an uneventful recovery. Immunoglobulin G antibody titer of the SARS-CoV by immunofluorescence assay was <1:25 on day 1 and 1:1,600 on day 39. In addition to CSF, RT-PCR for SARS-CoV was also positive in stool specimens and peritoneal fluid.

## Conclusions

In our patient, the occurrence of generalized convulsion with a positive RT-PCR for SARS-CoV in the CSF suggests possible infection of the central nervous system by SARS-CoV. Other possible causes of convulsion were considered. The slightly high serum sodium that gradually developed over 3 days was likely related to piperacillin/tazobactam administration and was unlikely to be responsible for the convulsion. Cerebral hypoxemia was unlikely, as the patient was monitored closely in the intensive care unit, and records did not show sustained arterial oxygen desaturation. Acute renal failure was also unlikely as the patient’s renal function was improving, and one would expect convulsion caused by acute renal failure to occur during the worsening phase or at the height of the renal impairment. Acid-base disturbances were absent. Although serum magnesium was somewhat low (0.69 mmol/L after correction for serum protein concentration using the formula suggested by Kroll and Elin [*4*]), convulsions are usually associated with much lower levels (*5*). No hypertension or proteinuria existed to suggest eclampsia. Also, preeclampsia occurs when the placenta is present, and termination of pregnancy is the standard treatment for severe preeclampsia (*6*). Ribavirin has not been reported to cause convulsion, and it would be very unlikely for such an event to occur 5 days after discontinuation.

The possibility of a false-positive RT-PCR test result was also considered. To our knowledge, a genuine false-positive test has not been reported in the literature in any clinical specimen. Nonetheless, the CSF sample could have been contaminated with the patient’s own blood, which contained genetic material of SARS-CoV. However, finding only 20 erythrocytes per mm^3^ in the CSF makes this unlikely.

Human coronavirus (HCoV) is responsible for up to one third of upper respiratory tract infections (*7*). It can enter susceptible cells through the endocytic pathway (*8*). Two strains (229E and OC43) have been implicated in multiple sclerosis (*9*,*10*), and both can persistently infect human oligodendrocytic and neuroglial cell lines (*11*). More recently, a combination of RT-PCR and Southern hybridization on human brain autopsy samples provided more definitive experimental evidence for the neurotropism and neuroinvasion of HCoV and its possible association with multiple sclerosis (*12*). Preliminary work in Hong Kong with RT-PCR on SARS autopsy specimens of brain tissue were positive for SARS-CoV, although electron microscopy did not show ultrastructural features of the virus (W.C. Yu, unpub. data). The findings from our patient are not compatible with multiple sclerosis, and the PCR result suggests that the central nervous system (CNS) is affected by SARS-CoV.

Another lumbar puncture cannot be repeated to test the CSF by RT-PCR. The presence of SARS-CoV in the CNS cannot be firmly established. The possibility also remains that infection of the CNS never occurred, as suggested by the lack of focal neurologic deficit, normal CSF pressure, cell count, and biochemistry. The normal electroencephalogram and magnetic resonance imaging might have missed the pathologic changes, as they were done 17 and 24 days after the event. In the absence of a good alternative explanation for the convulsion, the diagnosis of infection of the CNS by SARS-CoV is still possible, despite a lack of supportive evidence. The hospital has managed a total of 577 definite SARS patients in this period, and this is the only patient who had a convulsion. This was the only single patient who had a lumbar puncture, suggesting that the involvement of the CNS in SARS is rare.

Besides involvement of the lungs and possibly the CNS, no good alternative explanation exists for acute renal failure in this patient. Renal failure could possibly be caused by SARS-CoV involving the kidneys. Additionally, our patient had diarrhea from day 3 to day 20, with positive RT-PCR for SARS-CoV in stool specimens, suggesting involvement of the gastrointestinal tract as well. In conclusion, our case demonstrates that SARS-CoV can possibly infect multiple organ systems and that CNS can potentially be involved.

## References

[1] Booth CM, Matukas LM, Tomlinson GA, Rachlis AR, Rose DB, Dwosh HA, Clinical features and short-term outcomes of 144 patients with SARS in the greater Toronto area. JAMA. 2003;289:2801–9. 10.1001/jama.289.21.JOC30885 12734147

[2] Peiris JS, Chu CM, Cheng VC, Chan KS, Hung IF, Poon LL, Clinical progression and viral load in a community outbreak of coronavirus-associated SARS pneumonia: a prospective study. Lancet. 2003;361:1767–72. 10.1016/S0140-6736(03)13412-5 12781535 PMC7112410

[3] Lee N, Hui D, Wu A, Chan P, Cameron P, Joynt GM, A major outbreak of severe acute respiratory syndrome in Hong Kong. N Engl J Med. 2003;348:1986–94. 10.1056/NEJMoa030685 12682352

[4] Kroll MH, Elin RJ. Relationships between magnesium and protein concentrations in serum. Clin Chem. 1985;31:244–6. 3967355

[5] Dacey MJ. Hypomagnesemic disorders. Crit Care Clin. 2001;17:155–73. 10.1016/S0749-0704(05)70157-3 11219227

[6] Walker JJ. Pre-eclampsia. Lancet. 2000;356:1260–5. 10.1016/S0140-6736(00)02800-2 11072961

[7] Myint SH. Human coronavirus: a brief review. Rev Med Virol. 1994;4:35–46. 10.1002/rmv.1980040108

[8] Blau DM, Holmes KV. Human coronavirus HCoV-229E enters susceptible cells via the endocytic pathway. Adv Exp Med Biol. 2001;494:193–8. 11774468 10.1007/978-1-4615-1325-4_31

[9] Stewart JN, Mounir S, Talbot PJ. Human coronavirus gene expression in the brains of multiple sclerosis patients. Virology. 1992;191:502–5. 10.1016/0042-6822(92)90220-J 1413524 PMC7131143

[10] Salmi A, Ziola B, Hovi T, Reunanen M. Antibodies to coronaviruses OC43 and 229E in multiple sclerosis patients. Neurology. 1982;32:292–5. 6278362 10.1212/wnl.32.3.292

[11] Arbour N, Talbot PJ. Persistent infection of neural cell lines by human coronaviruses. Adv Exp Med Biol. 1998;440:575–81. 9782332 10.1007/978-1-4615-5331-1_75

[12] Arbour N, Day R, Newcombe J, Talbot PJ. Neuroinvasion by human respiratory coronaviruses. J Virol. 2000;74:8913–21. 10.1128/JVI.74.19.8913-8921.2000 10982334 PMC102086

